# Management of MASLD/MASH: challenges, innovations, and the future of patient-centered care in Japan

**DOI:** 10.1007/s00535-026-02401-9

**Published:** 2026-04-15

**Authors:** Yuya Seko, Kanji Yamaguchi

**Affiliations:** Molecular Gastroenterology and Hepatology, Graduate School of Medical Science, Kyoto Prefectural University of Medicine, 465 Kajii-Cho, Kawaramachi-Hirokoji, Kamigyo-Ku, Kyoto, 602-8566 Japan

**Keywords:** Fatty liver, Fibrosis, Japan, Metabolic dysfunction-associated steatotic liver disease, Metabolic dysfunction-associated steatohepatitis

## Abstract

Over the past decade, metabolic dysfunction-associated steatotic liver disease (MASLD) has become a leading cause of chronic liver disease in Japan, with estimated prevalence rising from 33.7% (2020) to 44.8% (2040), owing to an aging population and lean individuals. In this narrative review, we summarize the epidemiology, pathogenesis, diagnostic strategies, and emerging treatments for MASLD and metabolic dysfunction-associated steatohepatitis (MASH) in Japan, culminating in a proposed ideal patient care pathway tailored to local needs. MASLD pathogenesis involves complex interactions between metabolic dysfunction, inflammation, insulin resistance, and genetic susceptibility. In Japan, a stepwise diagnostic algorithm is endorsed, starting with non-invasive liver disease assessments (NILDA) such as fibrosis-4, followed by imaging-based fibrosis evaluation and selective biopsy. We propose a two-tiered diagnosis combining first-line NILDA (fibrosis-4) with second-line NILDA (e.g., enhanced liver fibrosis, Mac-2 binding protein glycosylation isomer, cytokeratin-18 fragment, and serum type IV collagen 7S) to refine risk stratification, reduce unnecessary referrals, and optimize healthcare resources, with qualifying patients referred to a hepatologist and treatment plans based on imaging-based NILDA or liver biopsy. The management of MASLD/MASH relies on lifestyle modification and pharmacological agents targeting metabolic comorbidities. However, novel anti-fibrotic, anti-inflammatory, and metabolism-targeted therapies are advancing, with future treatment decisions expected to integrate genomic and metabolomic profiling alongside NILDA. The proposed care model is multidisciplinary, engaging physicians, hepatologists, dietitians, psychologists, pharmacists, and hepatitis medical care coordinators to address both hepatic and systemic metabolic care. This evolving paradigm emphasizes proactive, personalized care informed by real-world data and long-term monitoring to improve outcomes and quality of life for patients with MASLD/MASH in Japan.

## Introduction

Over the past decade, the etiology of chronic liver disease in Japan has shifted dramatically. Historically, viral hepatitis was a leading cause of cirrhosis and hepatocellular carcinoma (HCC), both of which remain challenging to treat in their advanced stages. However, the prevalence of viral cirrhosis has declined due to the initiatives of national hepatitis control and the development of potent antivirals [1, 2]. In contrast, non-viral causes of cirrhosis are increasing, with alcoholic steatohepatitis being the leading etiology and a rising burden of metabolic dysfunction-associated steatotic liver disease/steatohepatitis (MASLD/MASH); these are new terms introduced in 2023 to reflect their metabolic origins [1–5].

In Japan, the prevalence of MASLD in individuals aged over 50 years was reported to be 25.9% in 2019. Additionally, lean MASLD accounted for 20.7% of the MASLD population [6]. Approximately 9–30% of Japanese adults have ultrasonography-diagnosed MASLD, with at least 10–20% diagnosed with MASH [7], indicating limited awareness and understanding of MASLD/MASH among clinicians and the general population.

Recently, the diagnosis and management of MASLD/MASH have advanced, with non-invasive liver disease assessment (NILDA), such as high-resolution imaging and blood-based biomarkers, enabling early diagnosis [8, 9]. The Food and Drug Administration (FDA) has recently announced that liver stiffness measurement (LSM) by vibration-controlled transient elastography (VCTE) is under evaluation as a potential surrogate endpoint in place of liver biopsy, and NILDA is expected to become increasingly important in the future. Emerging therapies for MASH, including lifestyle interventions and drug treatment with resmetirom and semaglutide, are reshaping therapeutic strategies [10, 11]. However, challenges such as the lack of standardized guidelines, biological variability, and clinical practice differences persist [12]. Therefore, a personalized treatment strategy is crucial within this therapeutic landscape. Hence, we conducted a literature review on the current scenario of MASLD/MASH diagnosis and management in Japan.

This narrative review focuses on recent research on the epidemiology, diagnosis, and treatment of MASLD/MASH in Japanese patients, an aging population with a low obesity rate. Additionally, it addresses disease heterogeneity in pathogenesis, risk prediction, and metabolism-based treatment response. Considering the distinctive social systems in Japan, such as the healthcare insurance system and health checkups, we believe that this review can provide insights into the unique features of MASLD/MASH in the Japanese population and support evidence-based management among Japanese clinicians. Since MASLD and non-alcoholic fatty liver disease (NAFLD) are known to have a high concordance [13], this paper avoids term duplication by interpreting references to NAFLD and non-alcoholic steatohepatitis (NASH) in the original articles as MASLD and MASH, respectively.

## Global and Japanese epidemiology of MASLD and MASH

**Fig. 1:**
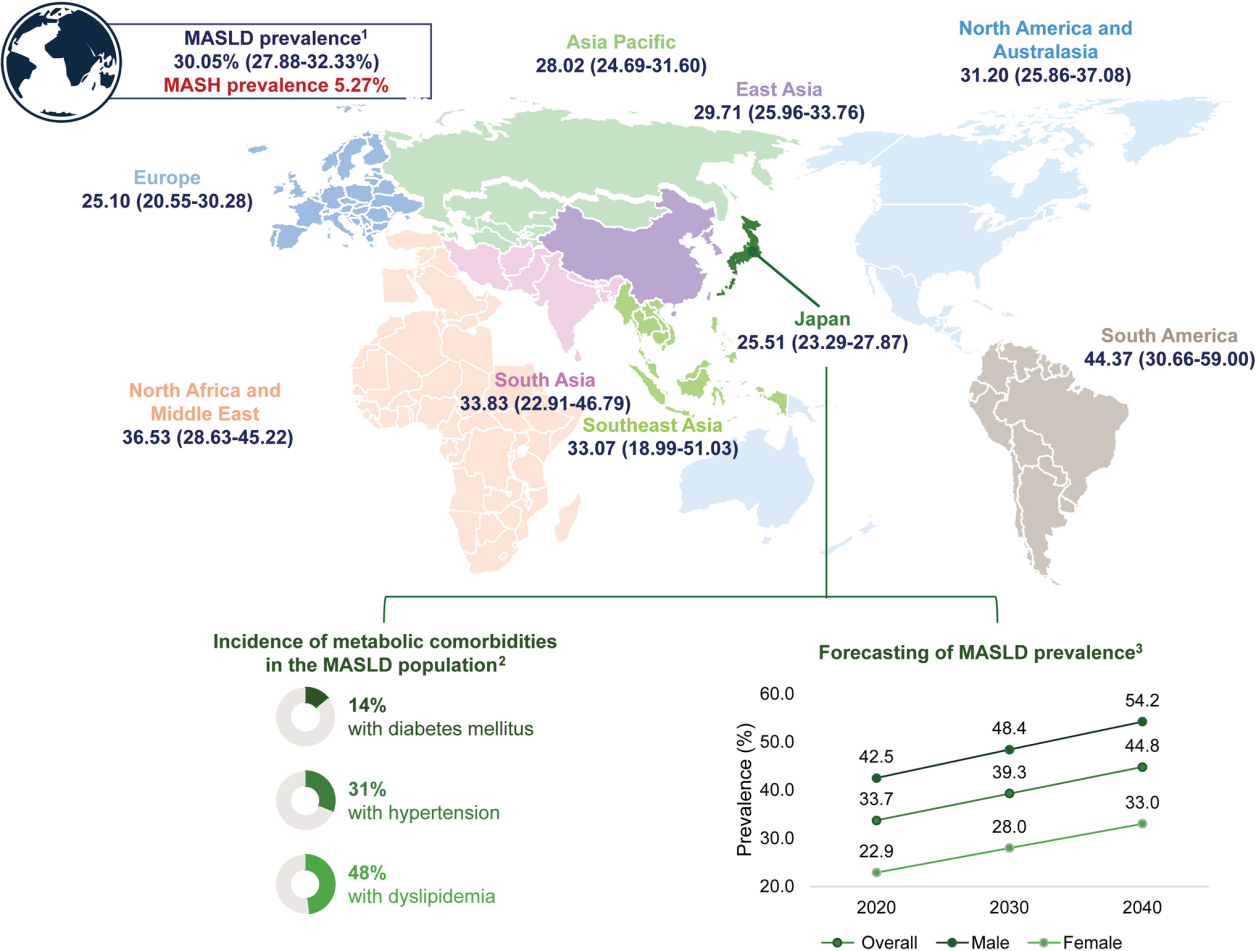
Epidemiology of MASLD: global and regional prevalence with a focus on Japan, future projections, and associated metabolic comorbidities. All values are in percentages. ^1^Younossi et al. (2023) Hepatology 77:1335–1347; ^2^Fujii et al. (2023) Hepatology Research 53:1059–1072; ^3^Ito et al. (2021) Hepatol Int 15:366–379. *MASH *metabolic dysfunction-associated steatohepatitis; *MASLD* metabolic dysfunction-associated steatotic liver disease. *MASH*

Globally, MASLD is the most common chronic liver disease, with a 24.3% increase in age-standardized prevalence and a 5.5% increase in both deaths and disability-adjusted life-years from 1990 to 2021 [14]. Global MASLD prevalence is 30.05% (27.88–32.33%), with the highest rates in South America (44.37%), North Africa and the Middle East (36.53%), South Asia (33.83%), Southeast Asia (33.07%), North America and Australasia (31.20%), East Asia (29.71%), Asia Pacific (28.02%), and Western Europe (25.10%) [15] (Fig. 1).

In Western countries, MASLD affects 24.1–30.5% of the population (1989–2015), while in Asia, the prevalence is 27.4–29.6% (1989–2019) [16, 17]. Among Asian countries, Japan has a relatively low prevalence (22.22% in 2005), potentially due to its traditional diet, although this increased to 29.61% by 2016 with westernization [6]. Regional variation exists within Japan, from 20.37% (Chubu) to 31.14% (Shikoku) [6].

Forecast analysis predicts a rise in prevalence from 33.7% (2020) to 41.4% by 2050 in the United States [18] and from 33.7% (2020) to 44.8% by 2040 in Japan [6] (Fig. 1). MASLD prevalence (fiscal year 2018–2019) in Japan increased from 21.8 to 30.3% in men and from 10.4 to 16.1% in women; notably, rates were highest in middle-aged men (22.9–32.5%) and older women (13.2–25.1%) [19]. MASLD prevalence varies by age, sex, body mass index (BMI), drinking and smoking status, and region [6, 20]. A large Japanese study found that MASLD patients had a 2.9-fold higher incidence of cardiovascular disease (CVD) (2.82 *vs* 0.97 per 1000 person years), along with a greater prevalence of hypertriglyceridemia and diabetes [21]. Among patients with MASLD from the medical health checkup registry for metabolic syndrome, chronic kidney disease, and fatty liver in Japan, 14% had diabetes mellitus, 31% had hypertension, and 48% had dyslipidemia [22] (Fig. 1).

The worldwide prevalence of MASH has also risen by 3–5% in parallel with increases in obesity, type 2 diabetes (T2D), and metabolic syndrome [15, 23]. In 2019, the highest prevalence was in South America (7.1%) and the lowest in Western Europe (4.0%) [15]. Among biopsied MASLD patients, MASH prevalence was significantly higher (59.1%), with 63.5% in Asia and 60.6% in North America [16]. In Japan, among 2012 obese MASLD patients, 349 (17.3%) had biopsy-confirmed MASH, while among 1603 lean MASLD patients, 61 (3.8%) had MASH [6].

## MASLD in lean individuals

Obesity is a major risk factor for MASLD, yet lean individuals are also affected. Globally, lean MASLD (BMI: < 23 kg/m^2^ [Asia], < 25 kg/m^2^ [United States]) accounts for 1.3–16% of the population, with particularly high prevalence in Asia [24]. In the United States, lean MASLD (BMI < 25 kg/m^2^) affects about 7%, while in Asia (BMI < 23 kg/m^2^), it affects 4–22.4% [25, 26]. In Japan, 20.7% of MASLD patients are lean (BMI < 23 kg/m^2^), with regional variation (6.1–22.8%) likely reflecting lifestyle and dietary differences [6]. Data from the Japanese NAGALA cohort (*n* = 15,299; 2004–2015) showed a MASLD prevalence of 14.5%, with 13.7% among non-obese (BMI < 30 kg/m^2^), non-diabetic individuals [14]. Lean MASLD (BMI < 25 kg/m^2^) is associated with a higher risk of cirrhosis and mortality compared with non-lean MASLD [27, 28]. In the National Health and Nutrition Examination Survey III, lean MASLD patients (BMI < 25 kg/m^2^) had higher all-cause (40.9% *vs* 17.9%) and cardiovascular (15.1% *vs* 3.7%) mortality *vs* non-lean individuals without MASLD (all *p* < 0.001) [28].

A study evaluating 466 MASLD patients reported significantly higher prevalence of lobular inflammation (16.2% *vs* 7.8%; *p* = 0.011), hepatocellular ballooning (25.7% *vs* 15.7%; *p* = 0.014), portal and periportal fibrosis (25.7% *vs* 13.3%; *p* = 0.019), progression to cirrhosis (8.1% *vs* 1.7%; *p* = 0.010), and MASH (18.9% *vs* 8.3%; *p* = 0.049) in lean (BMI ≤ 25 kg/m^2^) *vs* overweight (BMI > 25.0 to ≤ 30.0 kg/m^2^) patients [27]. Patatin-like phospholipase domain-containing protein 3 (*PNPLA3*) mutations, particularly rs738409 (I148M), are more frequent and severe in lean MASLD (BMI < 25 kg/m^2^) and may contribute to disease onset [29]. A Japanese study in MASLD patients (134 non-obese [BMI < 25 kg/m^2^] and 406 obese [BMI ≥ 25 kg/m^2^]) showed a higher *PNPLA3* rs738409 GG genotype (47.8% *vs* 36.5%; *p* = 0.02) and lower histological scores in non-obese than obese MASLD patients, with a strong association of the GG genotype in the non-obese group (odds ratio [OR]; 95% confidence interval [CI] 4.15; 2.68–6.43 *vs* 2.76; 1.77–4.31) [30]. Additionally, the GG genotype was associated with lobular inflammation (OR; 95% CI 3.37; 1.43–7.96 *vs* 0.99; 0.65–1.53; *p* = 0.0055), ballooning (2.51; 1.09–5.76 *vs* 1.46; 0.75–2.84; *p* = 0.031), and NAFLD activity score (NAS) (2.42; 1.09–5.40 *vs* 1.55; 1.00–2.40; *p* = 0.031) in non-obese MASLD [30]. This evidence highlights the ethnicity-specific characteristics of lean MASLD in Japan, emphasizing the importance of tailored approaches.

## Pathogenesis of MASLD/MASH

MASLD/MASH is not just a liver-specific condition: it is a systemic metabolic disorder, linking obesity, T2D, insulin resistance, hypertension, CVD, inflammation, and lipid dysmetabolism, key contributors beyond a liver-specific condition [31, 32]. In Japan, MASLD affects 25.8% of the general population according to the MIRACLE-J study, with comorbidities including dyslipidemia (48%), hypertension (31%), and/or diabetes mellitus (14%) [22]. The study also reported a positive correlation between MASLD prevalence and metabolic abnormalities, even without obesity, and estimated that 1–2% of cases have advanced fibrosis. Thus, MASLD is best understood as hepatic steatosis associated with metabolic dysfunction.

**Fig. 2:**
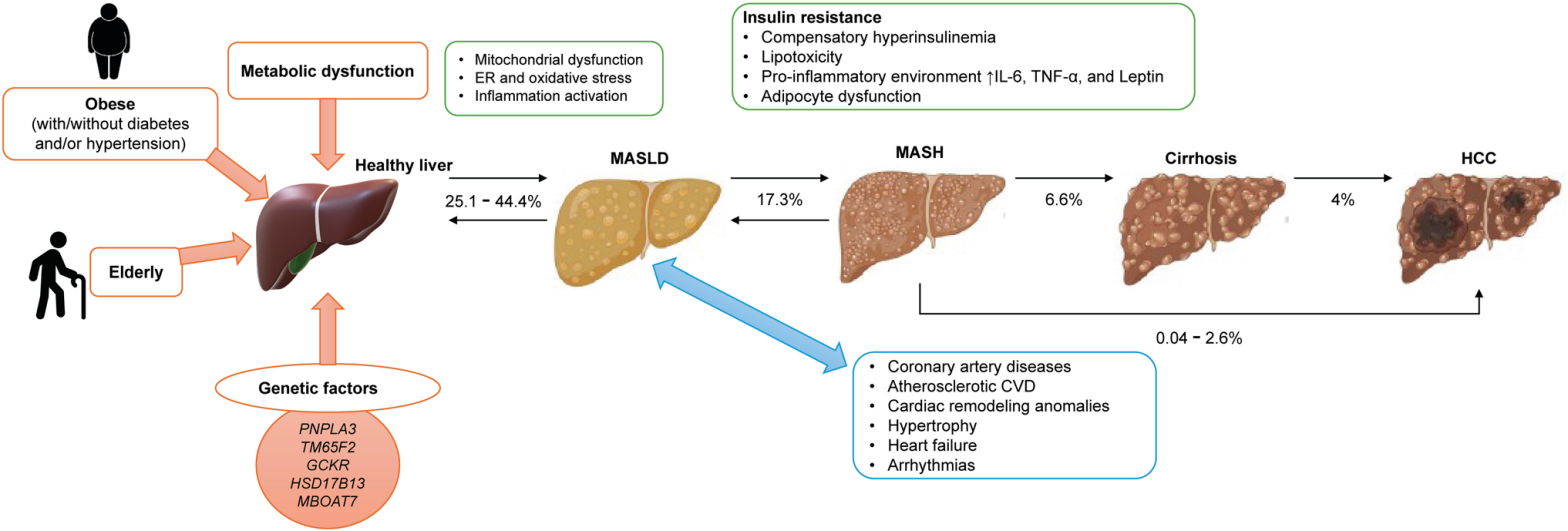
Pathogenesis and progression of MASLD/MASH-HCC. *CVD* cardiovascular disease, *ER* endoplasmic reticulum, GCKR glucose kinase regulator, *HCC* hepatocellular carcinoma, *HSD17B13* hydroxysteroid 17-beta dehydrogenase 13, *IL* interleukin, *MASH* metabolic dysfunction-associated steatohepatitis, *MASLD* metabolic dysfunction-associated steatotic liver disease, *MBOAT7* membrane-bound O-acyltransferase domain-containing 7, *PNPLA3* patatin-like phospholipase domain-containing protein 3, *TM6SF2* transmembrane 6 superfamily member 2, *TNF* tumor necrosis factor

Japanese guidelines suggest that distinct genetic and metabolic factors may influence disease progression [7, 33]. Overall, the pathogenesis of MASLD involves multiorgan interactions, with obesity and insulin resistance, inflammation and metabolic dysfunction, and genetic predisposition playing key roles in the initiation and progression of MASLD/MASH (Fig. 2).

## Obesity and insulin resistance

The pathogenesis of MASLD/MASH is driven by insulin resistance and lipotoxicity. Excess free fatty acids (FFAs) overload the adipose tissue, causing insulin resistance with compensatory hyperinsulinemia from pancreatic β-cells. Adipocyte dysfunction promotes FFA release to other organs, including the liver, muscle, and pancreas, while establishing a pro-inflammatory environment characterized by elevated interleukin-6 (IL-6), tumor necrosis factor alpha (TNF-α), leptin, and reduced adiponectin levels [31]. The liver becomes overwhelmed with increased FFA delivery and de novo lipogenesis, leading to lipotoxicity, mitochondrial dysfunction, endoplasmic reticulum stress, oxidative stress, and inflammasome activation, all of which drive hepatocyte injury and apoptosis [31]. A study in Japan showed that fasting insulin secretory function declines with the development of liver fibrosis in MASLD, indicating impaired β-cell function as a feature of liver fibrosis in relatively non-obese patients [34].

## Inflammation and metabolic dysfunction

MASLD represents a spectrum of liver disorders from benign steatosis to MASH, driven by molecular, metabolic, and cellular dysregulation that alters liver pathophysiology [35]. MASLD begins with intrahepatic fat accumulation (> 5% liver weight), triggered by insulin resistance, obesity, or dyslipidemia [36]. Excess FFAs from adipose lipolysis and de novo lipogenesis exceed hepatic β-oxidation, leading to triglyceride storage in lipid droplets and hepatic steatosis [35]. FFAs and impaired triglyceride export trigger a proinflammatory cascade (IL-6, TNF-α, and IL-1β), sustaining hepatic inflammation and cellular injury. In obesity and T2D, adipose dysfunction amplifies FFAs and cytokine levels, creating a feedforward loop that accelerates MASH progression [35].

The transition to MASH involves inflammation, fibrosis, and risk of carcinogenesis. Obesity, in combination with genetic polymorphisms in *PNPLA3*, transmembrane 6 superfamily member 2 (*TM6SF2*), glucokinase regulator (*GCKR*), hydroxysteroid 17-beta dehydrogenase 13 (*HSD17B13*), and membrane-bound O-acyltransferase domain-containing 7 (*MBOAT7*), increases the risk of advanced fibrosis and HCC [35]. Cirrhosis remains the most critical HCC risk factor, increasing malignancy risk by > 10-fold. Chronic inflammation disrupts genomic stability, telomere maintenance, and DNA damage responses, promoting HCC development [35].

In MASH, the severity of hepatic fibrosis is strongly linked to a broad spectrum of cardiovascular complications, including coronary artery disease, atherosclerosis, cardiac remodeling, hypertrophy, heart failure, and arrhythmia, likely driven by the liver’s central role in systemic glucose and lipid homeostasis [32]. A Japanese retrospective study reported yearly incidences of 1.04% for CVD, 0.83% for non-liver malignancies, and 0.30% for liver-related events [37].

## Genetic factors

Genetic predisposition contributes to MASLD/MASH pathogenesis, notably polymorphisms in *PNPLA3*, *TM6SF2*, *GCKR*, *HSD17B13*, and *MBOAT7* that are linked to disease development and progression, via disruption of glucose and lipid homeostasis [38]. MASLD shows heritability (35–61%), with first-degree relatives having up to a 12-fold increased risk than the general population [38].

### PNPLA3

The *PNPLA3* rs738409 c.444 C > G p.I148M is a key variant associated with MASLD (OR: 1.91), MASH (2.54), and HCC (2.68–5.9) [38–40]. *PNPLA3* I148M correlates with hepatic steatosis, fibrosis, lobular inflammation, ballooning, and NAS, as well as decreased serum triglycerides in Japanese MASLD patients [41]. In these patients, the *PNPLA3* genotypes—CG (OR: 2.33; 95% CI 1.37–3.93) and GG (3.51; 2.09–5.88)—were strongly associated with advanced fibrosis and linked to liver-related events including HCC (CG: 13.24; 1.80–97.24; GG: 22.26; 3.07–161.46) [42, 43]. Genome-wide association studies also identified *PNPLA3* rs2896019 as a risk variant for MASLD (OR: 1.85; 95% CI 1.67–2.05) and MASH-HCC (3.37; 2.21–5.14) [44].

### TM6SF2

The *TM6SF2* rs58542926 c.499 G > A p.E167K variant impairs hepatic very low-density lipoprotein secretion, promoting triglyceride and liver fat accumulation, steatosis, necroinflammation, ballooning, and fibrosis. It is associated with MASLD (OR: 1.82), MASH (1.37), advanced fibrosis (2.08), and HCC (1.72) [38–40]. In Japanese patients, the variant correlated with increased plasma triglyceride levels (*p* = 0.0009), increased MASLD risk (OR: 1.68; 95% CI 1.29–2.20), advanced fibrosis (1.49; 1.05–2.13), and liver-related events, including HCC (2.42; 1.50–3.90) [42, 45].

### GCKR

*GCKR*, a fructose-6-phosphate-dependent inhibitor of glucokinase involved in de novo lipogenesis regulation, is elevated in patients with MASLD. The *GCKR* rs780094 T allele is associated with hepatic steatosis and elevated alanine aminotransferase (ALT) (OR: 1.30; 95% CI 1.03–1.65) [46]. The association of *GCKR* rs1260326 with an increased risk of MASLD (OR: 1.38; 95% CI 1.25–1.53 to 1.49; 1.09–2.05), MASH (1.55; 1.10–2.17), and HCC (1.84; 1.23–2.75) has been reported in Japanese patients [39, 44].

### HSD17B13

*HSD17B13*, involved in steroid hormone signaling and lipid droplet remodeling, has a splice variant rs72613567 c.704/812 + 2dup (T > TA insertion) associated with decreased risk of MASLD (OR: 0.84), MASH (0.86), and HCC (0.67–0.77) [39]. In Japanese patients, rs72613567 was associated with reduced risk of MASLD and advanced fibrosis (OR: 0.70; 95% CI 0.53–0.94), and liver-related events, including HCC (0.77; 0.49–1.21) [42]. It mitigates liver injury in *PNPLA3* I148M carriers and is associated with reduced *PNPLA3* mRNA expression, suggesting a potential for targeted *HSD17B13* inhibition in this subpopulation [39, 47].

### MBOAT7

The *MBOAT7* rs641738 g.54173068 C > T, affecting transmembrane channel-like 4 (rs641738 c.50 G > A p.G17E), is associated with increased hepatic fat, liver damage, fibrosis, MASLD (OR: 1.42), MASH (1.18), advanced fibrosis (1.2), and HCC (2.10) [38–40].

## Testing for MASLD/MASH

The diagnosis of MASLD requires documented steatosis with at least one cardiometabolic risk factor. While liver biopsy remains the gold standard, its invasiveness, cost, complication risk, sampling variability, and patient reluctance limit its routine use [48]. NILDA, such as fibrosis-4 (FIB-4) and aspartate aminotransferase (AST) to Platelet Ratio Index has demonstrated clinical utility. Additionally, newer biomarkers, such as Mac-2 binding protein glycosylation isomer (M2BPGi), thrombospondin 2, and autotaxin, combined with ultrasonography and magnetic resonance elastography (MRE), have reportedly improved diagnostic accuracy [48]. In Japan, a stepwise diagnostic approach is recommended: blood-based tests, followed by imaging for fibrosis risk stratification (F0–F4; defined as F0, no fibrosis; F1, fibrosis stage ≥ 1; F2, fibrosis stage ≥ 2; F3, fibrosis stage ≥ 3; F4, fibrosis stage ≥ 4), and biopsy for uncertain or confirmatory cases [7, 33]. Table 1 summarizes blood-based markers and imaging for diagnosis and risk stratification.

**Table 1:** NILDA used for diagnosis and staging of steatosis, fibrosis, MASLD, and MASH in Japan

NILDA	Use	Strengths	Limitations	Cutoff	Cost (Yen)^1^	References (for cutoff)
*Blood-based markers*						
Pro-C3	Active fibrogenesis marker	Directly measures type III collagen synthesis for active fibrosis turnover, superior sensitivity for early-to-moderate fibrosis progression	Specialized laboratory test, influenced by systemic inflammation in diabetes/obesity patients	≥ F2: 10.781 ng/mL	N/A	[148]
COL4-7S	Fibrosis risk	Established marker rising reliably in advanced fibrosis/cirrhosis, routinely available and tracks longitudinal changes	Low specificity as also elevated in renal dysfunction or other fibrotic diseases, reduced sensitivity for early MASH in obese/diabetic cases, much of evidence is from Japan	F0: 3.4 ng/mL, F1: 3.8 ng/mL, F2: 3.9 ng/mL, F3: 4.4 ng/mL, F4: 6.3 ng/mL	1480	[55]
M2BPGi	Fibrosis risk	Exponential rise in advanced fibrosis; excellent for detection of cirrhosis, extensive clinical validation in viral hepatitis transferable to MASH	Background inflammation from diabetes/metabolic syndrome causes false positives, low sensitivity for early/moderate fibrosis stages, most of evidence is from Japan	≥ F2: 0.890 C.O.I	1940	[148]
Autotaxin	Fibrosis risk	Correlates with LPA-mediated fibrosis and HCC risk in advanced MASH, complements other markers for risk stratification	Fluctuates with obesity, diabetes, pregnancy, or cholestasis, most of evidence is from Japan	Liver-related events: 0.955 mg/L in men; 1.565 mg/L in women	1940	[62]
HA	Fibrosis risk	Extracellular matrix marker elevated in advanced fibrosis	Non-specific rise in joint disease, inflammation, or renal impairment, insensitive for early fibrosis detection	42 ng/mL	1790	[63]
CK-18F	Apoptosis marker	Quantifies hepatocyte apoptosis specific to MASH inflammation, reflects disease activity beyond steatosis alone	Non-specific elevation from extrahepatic cell death or drug injury, most of evidence is from Japan	At-risk MASH: ≥ 200 U/L	1940	[50]
*Imaging tests*						
FibroScan® (VCTE)	Liver stiffness	Rapid bedside test, validated staging with good reproducibility in ideal patients, non-invasive, rapid outpatient assessment of fibrosis (F2–F4 accuracy), abundant evidence for MASLD	Limited widespread inflammation/congestion falsely increases stiffness readings, failure rate increased in severe obesity (BMI > 30 kg/m^2^) or patients with narrow intercostal spaces	F2: 8.70 kPa; F3: 10.0 kPa; F4: 12.6 kPa	2000	[149]
FibroScan® (CAP)	Liver steatosis	Simultaneous steatosis quantification (S0–S3) during FibroScan, sensitive for early fat accumulation screening, bedside test, abundant evidence for MASLD	Unreliable in thick subcutaneous fat or deep chest wall, limited widespread	F2: 263.0 dB/m; F3: 283.0 dB/m; F4: 305.0 dB/m	2000	[149]
2D SWE	Liver stiffness	Rapid, bedside test, real-time liver stiffness mapping integrated with B-mode ultrasound, widely available	Limited evidence in MASLD, high operator dependence on probe pressure and ROI selection	F2: 8.7 kPa; F3: 9.1 kPa; F4: 11.6 kPa	2000	[150]
MRI-PDFF	Liver steatosis	Gold-standard quantitative fat measurement, precise for trials, minimal obesity interference, tracks treatment response accurately, considered the most accurate	Prohibitively expensive and time-intensive, limited availability	F2: 4.40%; F3: 11.7%; F4: 12.2%	6000	[149]
MRE	Liver fibrosis staging	Highest accuracy for whole-liver stiffness/fibrosis assessment, robust against obesity, superior to ultrasound methods	Requires specialized MRI hardware and expertise, limited availability	F2: 3.68; F3: 3.78; F4: 5.17 (presented as AUROC)	6000	[149]
*Scores and indices*						
FLI	Predictor of MASLD	Calculates steatosis risk from BMI, waist circumference, TG, and GGT, no extra tests needed, excellent for large-scale population screening, simple formula, cost effective	Overestimates prevalence in metabolic syndrome/obesity clusters, no information on fibrosis or MASH severity	FLI: ≥ 35 in men; ≥ 16 in women	N/A^2^	[66]
FIB-4	Fibrosis risk (serum index)	Simple score (age/AST/ALT/platelets) rules out advanced fibrosis effectively, zero added cost, ideal primary care triage, simple formula, cost effective	Age factor causes false positives in elderly, misses silent cases, low sensitivity in diabetic MASH with minimal ALT elevation	Low: < 1.3; Indeterminate: 1.3–2.67; High: > 2.67; Age-specific: ≤ 49 years: 1.05/1.21; 50–59 years: 1.24/1.96; 60–69 years: 1.88/2.67; ≥ 70 years: 1.95/2.67	N/A^3^	[70, 151]
ELF™	Fibrosis risk	Multi-marker panel (HA, PIIINP, TIMP-1) for robust moderate-severe fibrosis, higher accuracy than single markers across liver disease	Requires proprietary lab assay, higher costs, reduced liver specificity in systemic fibrosis from obesity/diabetes, not widely used yet	F2: 9.2 and 10.7; F3: 9.8 and 11.0; At-risk MASH: 8.9 and 10.8	1940	[54]

^1^Costs are shown only for tests currently available for routine clinical use in Japan; tests without listed costs are for research use only or are not currently available for clinical use

^2^Since the FLI is calculated from BMI and waist circumference, with GGT and TG, which are derived from routine blood tests, no extra cost was needed

^3^Since the FIB-4 is calculated from age, with AST, ALT, and platelets, which are derived from routine blood tests, no extra cost was needed

*ALT* alanine aminotransferase, *AST* aspartate aminotransferase, *AUROC* area under the receiver operating characteristic curve, *BMI* bodymass index, *CAP* controlled attenuation parameter, *CK*-*18F* cytokeratin-18 fragment, *C.O.I* cutoff point index, *COL4*-*7S* serum type IV collagen 7S, *ELF* enhanced liver fibrosis, *FIB*-*4* fibrosis-4 index, *FLI* fatty liver indicator, *F1* fibrosis stage ≥ 1, *F2* fibrosis stage ≥ 2, *F3* fibrosis stage ≥ 3, *F4* fibrosis stage ≥ 4, *GGT* gamma-glutamyl transferase, *HA* hyaluronic acid, *HCC* hepatocellular cancer, *LPA* lipoprotein-A, *M2BPGi* Mac-2-binding protein glycosylation isomer, MASH metabolic dysfunction-–associated steatohepatitis, *MASLD* metabolic dysfunction-associated steatotic liver disease, *MRE* magnetic resonance elastography, *MRI* magnetic resonance imaging, *MRI*-*PDFF* magnetic resonance imaging-proton density fat fraction, *N*/*A* not available, *NILDA* non-invasive liver disease assessment, *Pro*-*C3* pro-collagen III, *PIINP* procollagen II N-terminal propeptide, *ROI* region of interest, *2D SWE* two-dimensional shear wave elastography, *TG* triglycerides, *TIMP*-*1* tissue inhibitor of metalloproteinases-1, *VCTE* vibration-controlled transient elastography

### Blood-based biomarkers

Blood-based markers are essential for the non-invasive diagnosis and monitoring of MASLD/MASH. These biomarkers facilitate early detection of inflammation, fibrosis, and systemic metabolic complications, reducing reliance on liver biopsies. Pro-collagen III, a marker for extracellular matrix remodeling, can distinguish hepatic steatosis from MASH and reflect fibrogenesis severity [49]. Cytokeratin-18 fragment (CK-18F) is a blood-based apoptosis marker indicative of hepatocyte apoptosis and has been identified as a marker for MASH [50, 51]. In a Japanese cohort of biopsy-proven MASLD, CK-18F levels ≥ 278 U/L were an independent predictor of MASH (OR: 4.46; 95% CI 1.42–14.00). Furthermore, the FIC-22 score incorporating CK-18F levels and the FIB-4 index demonstrated high predictive accuracy for MASH (area under the receiver operating characteristic [AUROC]: 0.82) and advanced fibrosis (AUROC: 0.78) [52]. In Japanese patients with MASLD, a CK-18F level > 200 U/L identifies individuals at increased risk of at-risk MASH (stage F2), warranting hepatological evaluation, whereas CK-18F ≤ 200 U/L suggests a low likelihood of at-risk MASH and may be managed with routine follow-up [50] (Table 1).

Serum type IV collagen 7S (COL4-7S), a type IV collagen fragment, is a well-established blood-based marker of liver fibrosis [53]. Its levels increase with fibrosis progression in various liver diseases, including MASH. In Japanese biopsy-confirmed patients with MASLD, COL4-7S demonstrated high diagnostic accuracy for advanced fibrosis, regardless of T2D status (AUROC: 0.883 without diabetes *vs* 0.872 with diabetes), with AUROC values of 0.81 for fibrosis stage F2, 0.844 for fibrosis stage F3, and 0.743 for at-risk MASH [54]. A recent comparison by Ishiba et al. [54] showed that COL4-7S (a single marker) performed at least equivalently to the Enhanced Liver Fibrosis (ELF) score (a composite marker) in identifying advanced fibrosis in MASLD. Using chemiluminescent enzyme immunoassay (CLEIA), COL4-7S demonstrated strong diagnostic performance, with a cutoff value of ≥ 3.8 ng/mL indicating fibrosis stage F2 (sensitivity 77%, specificity 68%); at this threshold, sensitivity and specificity for fibrosis stage F3 were 91% and 64%, respectively. Median COL4-7S levels increased across fibrosis stages (F0-F4: 3.4, 3.8, 3.9, 4.4, and 6.3 ng/mL, respectively), and among evaluated fibrosis markers, COL4-7S showed the highest diagnostic accuracy, particularly for fibrosis stage F2 (AUROC: 0.795) [55].

M2BPGi is a validated biomarker in the Japanese population for diagnosing and staging MASLD, MASH, and liver fibrosis [56]. It outperforms liver biopsy in routine fibrosis staging and HCC risk stratification [56]. A meta-analysis of 24 studies, including Japanese patients, confirmed high sensitivity and specificity for advanced fibrosis (F3, F4), with optimal performance for cirrhosis (≥ F4; AUROC: 0.87) [57]. M2BPGi is now widely adopted in Japanese MASLD clinics, supported by established risk thresholds and insurance coverage for quantitative measurement (M2BPGi-Qt, AU/mL) [57, 58]. CLEIA enables precise and reproducible M2BPGi-Qt quantification, correlating well with existing methods [59]. In chronic liver disease, M2BPGi-Qt reflects both fibrosis and inflammation; incorporating inflammatory markers enhances its cirrhosis detection accuracy beyond M2BPGi-Qt alone [60].

Serum autotaxin levels correlate with liver inflammation and fibrosis severity in MASLD and other chronic liver diseases: despite lower overall diagnostic accuracy than MRE, they demonstrate high sensitivity for detecting fibrosis stages F2 and F3, supporting their use in identifying patients requiring further fibrosis evaluation [61]. In Japan, serum autotaxin measurement has been covered by National Health Insurance since 2018, with sex-specific cutoffs predicting liver-related events in MASLD of 0.955 mg/L in men (sensitivity 85.7%, specificity 90.0%) and 1.565 mg/L in women (sensitivity 69.2%, specificity 83.9%) [62].

Serum hyaluronic acid is an independent predictor of MASH and severe fibrosis in MASLD, with a cutoff of 42 ng/mL reliably excluding severe fibrosis (negative predictive value 100%; sensitivity 100%, specificity 89%) [63, 64].

### Hematological indices and scores

Indices such as the Fatty Liver Index (FLI) and Hepatic Steatosis Index (HSI), based on routine clinical and laboratory parameters, demonstrate good diagnostic accuracy for screening patients for MASLD (FLI: cutoff ≥ 35 in men; ≥ 16 in women; HSI: AUROC: 0.87) [65, 66]. In Japanese patients, high FLI was associated with hypertension, particularly in those with dysglycemia (hazard ratio: 2.05 in men; 2.98 in women) [67]. Overall, blood-based scores utilizing simple, inexpensive, and readily accessible parameters are invaluable for epidemiological and screening programs, particularly in settings where imaging modalities are impractical.

The FIB-4 index is a widely used, cost-effective, and simple NILDA for excluding advanced fibrosis, particularly in primary care settings and among non-hepatologists [68]. FIB-4, calculated using age, AST, ALT, and platelet count, identifies advanced fibrosis (F3/F4) in hepatitis C virus-mono-infected patients and predicts low liver-related risk in non-cirrhotic chronic hepatitis B virus infection [68]. In the Japanese population, FIB-4 demonstrated limited accuracy for MASLD detection, with area under the curve of 0.51 in men (sensitivity: 22.3%; specificity: 80.3%) and 0.53 in women (sensitivity: 86.2%; specificity: 20.0%) [66]. FIB-4 is also proposed as a prescreening tool to optimize referral and prioritize patients at risk for advanced liver diseases. Recent studies have refined its application in older adults (aged ≥ 65 years), where the standard 1.3 threshold retains a high negative predictive value for advanced fibrosis [69]. In a multicenter study, age-specific thresholds (≤ 49 years: 1.05/1.21; 50–59 years: 1.24/1.96; 60–69 years: 1.88/2.67; ≥ 70 years: 1.95/2.67) were shown to improve the diagnostic accuracy of advanced fibrosis in Japanese patients with MASLD [70], indicating a cutoff of 2.0 to be appropriate for patients aged ≥ 65 years. In Japanese MASLD patients, FIB-4 effectively predicted liver-related morbidity and events over 5 years [71]. Lastly, post hoc analyses showed that FIB-4 correlates with steatohepatitis activity, while NAS may enable monitoring of treatment response [72].

The ELF test, a non-invasive blood-based assay, measures biomarkers of liver extracellular matrix metabolism—hyaluronic acid, procollagen III amino-terminal peptide, and tissue inhibitor of matrix metalloproteinase 1—to assess fibrosis. For significant fibrosis (≥ F2), an ELF score ≥ 9.8 showed 70% sensitivity and 64% specificity, while for advanced fibrosis (≥ F3), an ELF score ≥ 10.5 demonstrated 67% sensitivity and 78% specificity [73]. In Japanese cohorts, the ELF test demonstrated higher AUROC values than FIB-4 across fibrosis stages, with sensitivity/specificity of 91.1%/50.8% (low cutoff) and 38.5%/92.8% (high cutoff) for detecting fibrosis stage F3. Combining ELF and FIB-4 further improved sensitivity to 98.5% [74]. ELF also outperformed FIB-4 in detecting cirrhosis [75].

Fibro-Scope, an AI/neural network-based tool, assesses liver fibrosis in MASH and MASLD using 11 routine clinical variables plus COL4-7S [76]. In biopsy-proven Japanese MASLD patients, combining FIB-4 with Fibro-Scope improved diagnostic and prognostic accuracy, particularly in the intermediate-risk group [77]. Additionally, it showed high performance in distinguishing fibrosis stages, with up to 99.5% sensitivity and 90.9% specificity for F0 *vs* F1-F4, and over 80% accuracy for F0-F1 *vs* F2-F4 using gray-zone analysis [78].

## Imaging-based tests

### Ultrasonography

Abdominal ultrasonography is a widely used, cost-effective, first-line diagnostic tool for MASLD. It detects increased hepatic echogenicity relative to the renal cortex and has good diagnostic performance for moderate-to-severe steatosis [79]. However, its accuracy is operator-dependent and subjective, declines with high BMI, and may fail in mild steatosis detection [80].

### VCTE

VCTE and ELF tests are used as second-line tools following the FIB-4 index, specifically in patients at intermediate (FIB-4: 1.3 to 2.67; NAFLD fibrosis score (NFS): −1.455 to 0.672) or high (FIB-4 > 3.25; NFS > 0.672) fibrosis risk. FibroScan®, a non-invasive device utilizing VCTE, quantitatively measures LSM to assess hepatic fibrosis. Despite guideline recommendations for its use in MASLD management [7, 33], its availability remains limited due to its high cost and confinement to specialties.

A 3-step diagnostic algorithm involving FIB-4 (step 1), ELF or COL4-7S (step 2), and VCTE (step 3) demonstrated higher specificity and positive predictive value for advanced fibrosis than a 2-step approach (FIB-4 + VCTE) in a cohort with MASLD, enhancing diagnostic accuracy and reducing unnecessary specialist referrals and liver biopsies [81]. Sequential use of FIB-4 and LSM-VCTE has a 9% false-negative rate for advanced fibrosis. Applying rule-out (< 1.3; < 8.0 kPa) and rule-in (≥ 3.48; ≥ 20.0 kPa) thresholds can reduce liver biopsy rates from 33% to 19–24% [82].

FibroScan^®^ also incorporates the controlled attenuation parameter (CAP), which enables real-time non-invasive estimation of hepatic fat content. CAP has demonstrated good diagnostic accuracy for steatosis grading [83]. In a Japanese cohort with MASLD, CAP showed an AUROC of 0.87 for steatosis detection, with an optimal cutoff of 256 dB/m [84].

### Shear wave elastography (SWE)

SWE is an advanced ultrasound-based imaging technique that quantitatively measures liver stiffness for the detection of fibrosis and steatosis severity. SWE measures shear wave velocity, which increases with liver stiffness and correlates with fibrosis severity [85]. In a broader meta-analysis, 2D-SWE showed lower area under the curve for significant fibrosis (0.75), advanced fibrosis (0.72), and cirrhosis (0.88) than VCTE, MRE, and point SWE, highlighting performance variability across populations and reference standards [86]. A recent meta-analysis reported a sensitivity of 0.85 and specificity of 0.72 for 2D-SWE in Japanese MASLD patients [87].

### MRI-estimated proton density fat fraction (MRI-PDFF)

Although not a fibrosis assessment tool, MRI-PDFF is a key non-invasive analytical method for quantifying liver steatosis, with superior accuracy, precision, and reproducibility compared with liver biopsy [88]. A meta-analysis of 38 studies reported AUROC values of 0.99 for ≥ S1, 0.89 for ≥ S2, and 0.90 for ≥ S3 steatosis, with strong performance in Asian populations [89]. MRI-PDFF is also a valuable biomarker in clinical trials, enabling patient stratification, longitudinal assessment, and treatment monitoring [88].

### MRE

MRE is the most accurate non-invasive imaging method for liver fibrosis assessment [90, 91]. It quantifies liver stiffness in the entire liver, is operator-independent, minimally affected by obesity, and highly reproducible, with AUROC values for detecting fibrosis of 0.772–0.869 for ≥ 1, 0.856–0.919 for ≥ 2, 0.870–0.981 for ≥ 3, and 0.882–0.993 for ≥ 4 [90]. The recommended thresholds for fibrosis staging range from 2.61 kPa (≥ 1) to 4.69 kPa (≥ 4) [90]. A meta-analysis reported MRE AUROC values of 0.88, 0.93, and 0.92 for stages 2, 3, and 4, respectively [92]. Another meta-analysis reported a sensitivity of 0.91 and specificity of 0.96 for MRE in Japanese MASLD patients [87]. MRE is increasingly recognized for monitoring disease progression and evaluating therapeutic responses in MASLD [92].

### Liver biopsy

Liver biopsy remains the “gold standard” for diagnosing MASH and staging fibrosis and is a preferred endpoint for clinical trials [7, 33, 93]. It provides detailed histology and prognostic information but is invasive, costly, and limited by sampling error and observer variability [80, 94]. However, its necessity has decreased with the MASLD framework and the widespread uptake of validated NILDA.

Despite their diagnostic advantages, advanced techniques are limited by cost and availability, particularly outside tertiary centers. These limitations affect scalability for population-level screening and use in low-resource settings. The sequence of NILDA should align with local healthcare infrastructure, ideally in consultation with hepatologists to ensure test selection, interpretation, and integration into clinical care.

## Current MASLD/MASH treatment and status of future treatment development

MASLD/MASH represents a growing global health challenge. Current management involves lifestyle interventions and off-label pharmacotherapy targeting metabolic comorbidities. While diet and exercise remain the mainstay for MASLD management, the therapeutic landscape is evolving for moderate-to-advanced MASH-associated fibrosis with the FDA approval of resmetirom, a β-selective thyromimetic, in 2024, and semaglutide, a glucagon-like peptide-1 receptor agonist (GLP-1RA), in 2025 [95]. Meanwhile, the evolution of novel anti-fibrotic targets holds promise for future treatment options.

### Treatment policy in overseas and Japanese guidelines

The American Association for the Study of Liver Diseases, the European Association for the Study of the Liver, and the Japanese Society of Hepatology provide evidence-based practice guidelines for MASLD/MASH management, all of which emphasize lifestyle modification as the foundation of care [4, 7, 33, 96]. In addition to lifestyle modification, pharmacotherapy may be recommended as needed. The Japanese guidelines, particularly, advocate a stratified approach: intensive lifestyle interventions in the absence of advanced fibrosis, and metabolic comorbidity management in the presence of fibrotic MASH (F2-F3) [7, 33].

### Diet

Dietary modification remains the cornerstone of MASLD/MASH management. Evidence underscores the role of specific eating patterns in disease development, including Japanese non-obese individuals [97]. A retrospective cross-sectional study reported that low fish intake and frequent consumption of preserved foods were associated with increased risk of non-obese MASLD in Japan [98]. Nationwide survey data from fiscal years 2008–2019 also showed a positive correlation between MASLD prevalence in Japan and intake of total fat, saturated and monounsaturated fatty acids, and a high n6/n3 polyunsaturated fatty acid ratio (all *p* = 0.0005) [99]. In a cross-sectional study of 136 Japanese patients with MASLD, greater adherence to a modified Japanese Diet Index was independently associated with lower fibrosis risk (Agile 3 +; OR: 0.77; 95% CI 0.61–0.99), while higher skeletal muscle mass (≥ 75th percentile) showed a strong inverse association (OR: 0.23; 95% CI 0.07–0.77); soybean intake was positively associated with skeletal muscle mass (OR: 1.02; 95% CI 1.00–1.04) [100]. In a population-based study of 727 adults, a vegetable-based Japanese diet was associated with reduced liver fibrosis risk in MASLD (OR: 0.38; 95% CI 0.16–0.88), with higher α-tocopherol intake conferring a protective effect (OR: 0.74; 95% CI 0.56–0.99) [101]. Overall, these findings highlight the protective role of traditional Japanese diets and skeletal muscle preservation as key factors in fibrosis risk reduction in MASLD.

Studies have shown that dietary modification, weight loss, and exercise improve liver function and slow disease progression in Japanese patients with MASLD/MASH. A prospective cohort study showed that sustained carbohydrate restriction (150–200 g/day) in MASLD patients achieved ALT normalization (60%) and ≥ 7% weight loss (49%) at 12 months, alongside improvements in liver and metabolic markers [102]. In another retrospective study, a 6-day inpatient diet and exercise program led to long-term improvement in liver enzymes and body weight compared with outpatient care [103]. These findings highlight the potential of culturally tailored dietary interventions to improve liver outcomes and slow disease progression in Japanese MASLD/MASH populations.

### Exercise

Exercise is equally critical in MASLD/MASH management. A retrospective cross-sectional study reported significantly lower physical activity in Japanese patients with non-obese MASLD, highlighting its association with disease risk [98]. A 3-month exercise program in obese Japanese men with MASLD showed reduced liver steatosis, stiffness, inflammation, and fibrosis markers [104]. A 10-year cohort study found that regular exercise was associated with MASLD remission in men but not in women, indicating sex-specific effects [105]. Additionally, a 71-year-old Japanese woman with MASLD stage F2 showed significant metabolic and fibrosis improvement after 60 weeks of daily low-intensity resistance exercise without other interventions [106]. These findings support tailored physical activity as a key component in MASLD/MASH management in Japan.

The Japan Society of Hepatology liver rehabilitation program, which aims to promote high-quality clinical research and strengthen the evidence base for its application, recommends a personalized diet and aerobic exercise program to reduce hepatic steatosis and fibrosis, and preserve skeletal muscle mass, including in lean and sarcopenic patients [107, 108].

## Current pharmacological treatment strategies

Current treatment strategies for MASLD/MASH focus on agents targeting key metabolic and fibrotic pathways [109, 110]. Recent evidence highlights promising drug classes under development, including thyroid hormone receptor (THR)-β agonists, GLP-1RA, sodium-glucose cotransporter 2 inhibitors (SGLT2i), dipeptidyl peptidase-4 inhibitors (DPP-4i), fibroblast growth factor 21 (FGF21) analogs, pan-peroxisome proliferator-activated receptors (PPAR) agonists, fatty acid synthase inhibitors, and farnesoid *X* receptor agonists. Management of metabolic comorbidities such as T2D, dyslipidemia, and hypertension remains crucial, given their role in disease progression and cardiovascular risk.

Semaglutide, a GLP-1RA, is the latest drug to be FDA-approved for MASH (August 2025), with several studies supporting the use of GLP-1RA and SGLT2i in MASLD/MASH patients in Japan [7, 33, 111]. Clinical trials of GLP-1RA, such as semaglutide, tirzepatide, efinopegdutide, and survodutide, demonstrated safety and efficacy in improving liver steatosis and resolving MASH [112–115]. A retrospective study showed that 48 weeks of SGLT2i therapy improved body weight, glycemic control, liver enzymes, and the FIB-4 index, with sustained anti-fibrotic effects over 3 years [116]. Dapagliflozin improved MASH and fibrosis outcomes without causing deterioration in either and outperformed placebo [117].

Recent evidence on DPP-4i in MASLD is limited, with variable findings regarding its impact [118]. FGF21 analogs, pegozafermin and efruxifermin, showed promising results in treating MASH in phase II trials, although phase III trials are ongoing. Pegozafermin significantly reduced hepatic fat and improved histology in 63% of patients, with confirmed fibrosis improvement [112, 119, 120]. Efruxifermin also led to notable reductions in hepatic fat and fibrosis over 24 weeks [121].

The pan-PPAR agonist lanifibranor demonstrated efficacy for MASLD/MASH in clinical trials. Lanifibranor reduced hepatic fat and improved insulin resistance in patients with T2D and achieved MASH resolution without worsening fibrosis [122, 123]. Pemafibrate, a selective PPARα modulator, significantly reduced MRE-based liver stiffness, with improvements in ALT, other liver enzymes, lipid parameters, and fibrosis-related biomarkers and with a favorable safety profile [124, 125]. PXL065, a deuterium-stabilized R-pioglitazone lacking PPARγ activity, showed ≥ 30% liver fat reduction and improved NAS, fibrosis stability, and metabolic markers [126].

Resmetirom, a THR-β agonist, is one of the FDA-approved drugs for MASH (March 2024), demonstrating improvements in liver histology and liver parameters in a phase II trial [127]. The MAESTRO-NAFLD-1 trial confirmed safety and sustained reductions in hepatic fat and atherogenic lipids over 52 weeks [95]. In the phase III trial, MAESTRO-NASH, resmetirom achieved MASH resolution and fibrosis improvement [11].

Statins have shown benefits in MASLD, including significant reductions in liver enzymes and improvement in lipid profiles [128]. A Japanese claims database study (*N* = 253,383 statin users; 2005–2020) showed that high adherence (proportion of days covered ≥ 0.80) to statin therapy, rather than dose intensity, is associated with reduced MASLD risk (OR: 0.82) [129].

Although no antihypertensive drugs have shown direct histological benefit, angiotensin II receptor antagonists and angiotensin II-converting enzyme inhibitors remain critical components of MASH management in patients with hypertension [7, 33].

## Emerging therapeutic agents targeting MASLD/MASH: mechanistic insights into fibrosis, inflammation, and metabolism

The evolving understanding of MASLD/MASH has driven drug development targeting key pathophysiological pathways. Emerging therapies aim to (1) prevent or reverse fibrogenesis, (2) reduce hepatic inflammation, and (3) improve metabolic regulation (Table 2), offering a comprehensive strategy to reduce disease progression and improve long-term outcomes.

**Table 2:** Key features of clinical trials targeting fibrosis, metabolic dysfunction, and inflammation in MASLD/MASH

Drug name	Mode of action	Target effects	Trial name	Phases	Trial number	Enrollment	Country/region	Time frame	Current status	Citation
*Anti-fibrosis*										
Rencofilstat	Cyclophilins	Fibrosis	ALTITUDE	Phase II	NCT05461105	60 (actual)	US	120 days	Completed	https://clinicaltrials.gov/study/NCT05461105 [130]
	Results of primary endpoint									
	Mean (SD) DuO’s disease severity index (DSI) decreased from baseline 16.92 (4.31) to 16.35 (4.58) at Day 60 and 16.43 (4.98) at Day 120; and portal–systemic shunting fraction (SHUNT%) decreased from 26.07 (7.32) at baseline to 24.44 (7.36) at Day 60 and 24.89 (7.46) at Day 120									
Tropifexor and cenicriviroc	FXR agonists and CCR2/CCR5 antagonists	Fibrosis	TANDEM	Phase II	NCT03517540	193 (actual)	US, Argentina, Belgium, Canada, Czechia, Egypt, France, Germany, India, Israel, Italy, Latvia, Russia, Singapore, Spain, Turkey, UK	48 weeks	Completed	https://clinicaltrials.gov/study/NCT03517540 [131]
	Results of primary endpoint									
	Safety and tolerability: Any AE (%): TXR_140_, 84.0%; CVC, 85.4%; TXR_140_ + CVC, 85.1%; and TXR_90_ + CVC, 87.5%. SAEs (%): TXR_140_, 10.0%; CVC, 6.3%; TXR_140_ + CVC, 8.5%; and TXR_90_ + CVC, 20.8%. AEs leading to discontinuation (%): TXR_140_, 18.0%; CVC, 6.3%; TXR_140_ + CVC, 17.0%; and TXR_90_ + CVC, 2.1%. TEAE ≥ 10% in any group (%): pruritus: TXR_140_, 40.0%; CVC, 20.8%; TXR_140_ + CVC, 31.9%; and TXR_90_ + CVC, 20.8%									
Cenicriviroc	CCR2 and CCR5 antagonism	Fibrosis	CENTAUR	Phase II	NCT02217475	289 (actual)	US, Australia, Belgium, France, Germany, Hong Kong, Italy, Poland, Spain, UK	2 years	Completed	https://clinicaltrials.gov/study/NCT02217475 [132, 133]
	Results of primary endpoint									
	≥ 1-stage fibrosis improvement (regardless of MASH status): Year 1: CVC, 27.7% vs placebo, 16.7%; Year 2: CVC, 22.2% vs placebo, 19.3%. ≥ 1-stage fibrosis improvement with no worsening of MASH: Year 1: CVC, 19.9% vs placebo, 11.1% (OR: 2.03; 95% CI 0.89–4.62; *p* = 0.09); Year 2: CVC 12.8% vs placebo 14.0% (OR: 1.15; 95% CI 0.48–2.75; *p* = 0.75)									
Ertugliflozin	SGLT2 inhibitors	Fibrosis	Ertu-NASH	Phase IV	NCT05644717	164	Pakistan	24 weeks	Ongoing	https://clinicaltrials.gov/study/NCT05644717
GFT505 (Elafibranor)	PPAR agonists	Fibrosis		Phase IIb	NCT01694849	275 (actual)	US, Belgium, France, Germany, Italy, Netherlands, Romania, Spain, UK	52 weeks	Completed	https://clinicaltrials.gov/study/NCT01694849 [152]
	Results of primary endpoint									
	Improvement in NAS: NAS ≥ 4 (moderate and severe): Elafibranor80, 20%; Elafibranor120, 20%; placebo, 11% (OR: 3.16; 95% CI 1.22–8.13; *p* = 0.018). NAS 3 (mild): Elafibranor80, 40%; Elafibranor120, 29%; placebo, 50%									
Losartan	ARBs	Fibrosis	FELINE	Phase III	NCT01051219	45 (actual)	UK	2 years	Completed	https://clinicaltrials.gov/study/NCT01051219
Selonsertib	ASK1	Fibrosis		Phase II	NCT02466516	72 (actual)	US, Canada	28 weeks	Completed	https://clinicaltrials.gov/study/NCT02466516
	Results of primary endpoint									
	Safety and tolerability: Any TEAE (%): SEL 6 mg, 85.0%; SEL 18 mg, 68.2%; SEL 6 mg + SIM 125 mg, 90.0%; SEL 18 mg + SIM 125 mg, 90.0%; and SIM 125 mg, 70.0%. SAEs (%): SEL 6 mg, 10.0%; SEL 18 mg, 9.1%; SEL 6 mg + SIM 125 mg, 0%; SEL 18 mg + SIM 125 mg, 10.0%; and SIM 125 mg, 0%. Any grade ≥ 1 laboratory abnormality (%): SEL 6 mg, 85.0%; SEL 18 mg, 95.5%; SEL 6 mg + SIM 125 mg, 90.0%; SEL 18 mg + SIM 125 mg, 100.0%; SIM 125 mg, 80.0%									
Aldafermin	Activation of FGFR1c-KLB	Fibrosis	ALPINE 2/3	Phase IIb	NCT03912532	171 (actual)	US, Puerto Rico	24 weeks	Completed	https://clinicaltrials.gov/study/NCT03912532 [153]
	Results of primary endpoint									
	Liver fibrosis response (Mean NAS score [SE]): Aldafermin 0.3 mg, 0.24 [0.04]; Aldafermin 1.0 mg, 0.25 [0.04]; Aldafermin 3.0 mg, 0.27 [0.07]; placebo, 0.23 [0.06]. Safety and tolerability: Any TEAE (%): Aldafermin 0.3 mg, 69.8%; Aldafermin 1.0 mg, 82.9%; Aldafermin 3.0 mg, 88.4%; placebo, 83.7%. Any aldafermin-related TEAE (%): Aldafermin 0.3 mg, 30.2%; Aldafermin 1.0 mg, 48.8%; Aldafermin 3.0 mg, 48.8%; placebo, 32.6%. Any serious TEAE (%): Aldafermin 0.3 mg, 2.3%; Aldafermin 1.0 mg, 9.8%; Aldafermin 3.0 mg, 2.3%; placebo, 7.0%									
PF-06865571	DGAT2 inhibitors	Fibrosis	MIRNA	Phase II	NCT04321031	256 (actual)	US, Bulgaria, Canada, China, Hong Kong, India, Japan, Korea, Poland, Puerto Rico, Slovakia, Taiwan	48 weeks	Completed	https://clinicaltrials.gov/study/NCT04321031 [154]
	Results of primary endpoint									
	Resolution of MASH without worsening/≥ 1-stage fibrosis improvement without worsening of MASH/both (Mean, 90% CI) PF-06865571 25 mg BID, 0.45 (0.38–0.53); PF-06865571 75 mg BID, 0.48 (0.42–0.55); PF-06865571 150 mg BID, 0.50 (0.43–0.57); PF-06865571 300 mg BID, 0.51 (0.43–0.59); placebo 0.38 (0.26–0.50)									
Nitazoxanide	PFOR enzyme	Fibrosis		Phase II	NCT03656068	21 (actual)	US	28 weeks	Completed	https://clinicaltrials.gov/study/NCT03656068
	Results of primary endpoint									
	Safety and tolerability, n (%): Any TEAE: 20 (95.2%); maximum TEAE severity grade 3 (severe): 3 (14.3%); any treatment-related TEAE: 18 (85.7%); any SAE: 4 (19.0%); and any study-drug–related SAE: 0 (0.0%)									
Cotadutide	GLP-1RA	Fibrosis	PROXYMO-ADV	Phase II	NCT05364931	54 (actual)	US, Argentina, Australia, Austria, Canada, France, Germany, Greece, Israel, Italy, Japan, Korea, Malaysia, New Zealand, South Africa, Spain, Taiwan, Thailand, Turkey, UK	28 days	Completed	https://clinicaltrials.gov/study/NCT05364931
	Results of primary endpoint									
	Safety and tolerability: Any AE (%): Cotadutide 300 µg, 94.1%; Cotadutide 600 µg, 88.9%; placebo, 68.4%; Abnormal vital signs (%): Cotadutide 300 µg, 76.5%; Cotadutide 600 µg, 77.8%; placebo, 89.5%; Abnormal laboratory assessments (%): Cotadutide 300 µg, 94.1%; Cotadutide 600 µg, 94.4%; placebo, 100.0%; Treatment-emergent abnormal 12-lead ECG (%): Cotadutide 300 µg, 23.5%; Cotadutide 600 µg, 11.1%; placebo, 31.6%; Treatment-induced ADA (%): Cotadutide 300 µg, 41.2%; Cotadutide 600 µg, 61.1%; placebo, 0.0%; ADA titer, median (range): Cotadutide 300 µg, 240 (15–7680); Cotadutide 600 µg, 60 (15–240)									
GB1211	Gal-3 inhibitor	Fibrosis		Phase I	NCT03809052	78 (actual)	UK	7 weeks	Completed	https://clinicaltrials.gov/study/NCT03809052 [155]
	Results of primary endpoint									
	Safety and tolerability: AEs (%): A1-5 mg GB1211, 50.0%; A2-20 mg, 16.7%; A3-50 mg food-effect, 16.7%; A4-100 mg, 16.7%; A5-200 mg, 0.0%; A6-50 mg, 33.3%; A7-400 mg, 16.7%; Part A placebo, 35.7%; B1-50 mg BID, 12.5%; B2-100 mg BID, 50.0%; and Part B placebo BID, 50.0%									
*Anti-inflammatory*										
CM-101		Anti-inflammatory		Phase I	NCT06037577	8 (actual)	Israel	10 weeks	Completed	https://clinicaltrials.gov/study/NCT06037577 [134]
	Results of primary endpoint									
	Safety and tolerability: Any TEAE (%): IV CM-101 2.5 mg/kg, 33.3%; IV placebo, 50.0%; SC CM-101 5.0 mg/kg, 83.3%; and SC placebo, 0.0%. Serious TEAEs (%): IV CM-101 2.5 mg/kg, 0.0%; IV placebo, 0.0%; SC CM-101 5.0 mg/kg, 16.7%; and SC placebo, 0.0%. PK summary: IV CM-101 showed biphasic PK with slow distribution and elimination (t½ ~ 20.3 days). SC CM-101 (5.0 mg/kg) showed moderate-high interpatient variability, no steady state by dose 5, and a shorter terminal half-life (t½ ~ 16.2 days)									
Salsalate	Inhibition of COX	Anti-inflammatory		Phase IV	NCT03222206	34 (actual)	Korea	8 weeks	Completed	https://clinicaltrials.gov/study/NCT03222206
Aspirin	Inhibition of COX	Anti-inflammatory		Phase I/II	NCT04031729	80 (actual)	US	6 months	Completed	https://clinicaltrials.gov/study/NCT04031729 [135]
	Results of primary endpoint									
	Absolute change in intrahepatic lipid content (^1^H-MRS) (LSM [95% CI]) Aspirin, −6.6% (−11.9 to −1.3) vs placebo 3.6% (−1.7 to 8.9)									
Miricorilant	GR modulation and MR antagonism	Anti-inflammatory		Phase I	NCT05117489	70 (actual)	US	24 weeks	Completed	https://clinicaltrials.gov/study/NCT05117489
MN-001		Anti-inflammatory		Phase II	NCT02681055	19 (actual)	US	12 weeks	Completed	https://clinicaltrials.gov/study/NCT02681055
	Results of primary endpoint									
	Cholesterol efflux capacity (week 12): Mean change from baseline, −0.013 (SD 0.1022). Triglycerides (week 8): Mean change from baseline, −21.67 mg/dL (SD 27.89)									
Diacerein	Inhibition of IL-1β	Anti-inflammatory	DGCLFT2DM	Phase III	NCT02242149	84 (actual)	Brazil	24 months	Completed	https://clinicaltrials.gov/study/NCT02242149 [156]
	Results of primary endpoint									
	Hepatic biomarkers (during treatment; adjusted difference in mean change vs placebo): ALT, + 2 U/L (95% CI −3 to 8; *p* = 0.34); AST, + 1 U/L (−1 to 4; *p* = 0.34); GGT, 0 U/L (−9 to 9; *p* = 0.95); ALP, −4 U/L (−12 to 4; *p* = 0.33); and albumin, + 1 g/L (0 to 2; *p* = 0.16)									
*Metabolic dysfunction*										
Obeticholic acid	FXR agonists	Metabolic	CONTROL	Phase II	NCT02633956	84 (actual)	US	16 weeks	Completed	https://clinicaltrials.gov/study/NCT02633956 [157]
	Results of primary endpoint									
	LDL cholesterol (LSM change from baseline at week 16, mg/dL): Obeticholic acid 5 mg, −33.2 (SE 4.93); 10 mg, −44.27 (4.10); 25 mg, −39.54 (4.24); placebo, −53.33 (3.93)									
EDP-305	FXR agonists	Metabolic		Phase I	NCT03748628	8 (actual)	US	9 days	Completed	https://clinicaltrials.gov/study/NCT03748628
OKT3	Targeting CD3	Metabolic		Phase II	NCT01205087	36 (actual)	Israel	60 days	Completed	https://clinicaltrials.gov/study/NCT01205087
Metabolic cofactor supplementation, sorbitol		Metabolic		Phase II	NCT04330326	32 (actual)	Turkey	10 weeks	Completed	https://clinicaltrials.gov/study/NCT04330326 [158]
	Results of primary endpoint									
	Hepatic fat (MRI-PDFF): Reduced hepatic fat at Day 14: FC, 0.895; Padj = 0.096; a significant reduction at Day 70: FC, 0.887; Padj = 0.033; placebo showed no change at Day 14: FC, 0.963; Padj = 0.968 or Day 70: FC, 0.969; Padj = 0.825									
ALG-055009	THR-β agonists	Metabolic	HERALD	Phase IIa	NCT06342947	102 (actual)	US	12 weeks	Completed	https://clinicaltrials.gov/study/NCT06342947
Saroglitazar	PPAR agonists	Metabolic		Phase IIa	NCT03639623	20 (actual)	US	24 weeks	Completed	https://clinicaltrials.gov/study/NCT03639623 [159]
	Results of primary endpoint									
	Safety and tolerability: Any AE: Saroglitazar magnesium 4 mg, 12 participants (60.0%)									
Hcy- lowering supplements		Metabolic		Early phase I	NCT05720702	31 (actual)	US	12 weeks	Completed	https://clinicaltrials.gov/study/NCT05720702
ISIS 703802		Metabolic		Phase II	NCT03371355	105 (actual)	US, Canada	27 weeks	Completed	https://clinicaltrials.gov/study/NCT03371355
	Results of primary endpoint									
	Percent change in fasting triglycerides (geometric mean, 95% CI): ISIS 703802 40 mg Q4W, −36% (−47 to −23); 80 mg Q4W, −53% (−60 to −43); 20 mg QW, −47% (−57 to −35); placebo, −16% (−29 to 0)									
RO5093151	11β-HSD1 inhibition	Metabolic		Phase I	NCT01277094	82 (actual)	Austria, Germany	12 weeks	Completed	https://clinicaltrials.gov/study/NCT01277094 [160]
	Results of primary endpoint									
	Liver fat content, baseline to week 12: RO5093151, 16.75% (SD 8.67) to 14.28% (8.89); placebo, 18.53% (10.00) to 18.46% (10.78); between-group difference *p* = 0.02									
Volixibat (SHP626)	ASBT inhibition	Metabolic		Phase I	NCT02571192	8 (actual)	US	10 days	Completed	https://clinicaltrials.gov/study/NCT02571192 [161]
	Results of primary endpoint									
	PK and mass balance ([^14^C]-volixibat, single oral 50 mg): Plasma volixibat concentrations (0–0.179 ng/mL) up to 8 h post-dose, with no detectable radioactivity in plasma or whole blood. Urinary recovery (mean cumulative 0.01% of dose; 4423.5 ng). Fecal excretion recovery (mean 92.3% of dose)									
Resmetirom	THR-β agonists	Metabolic	MAESTRO-NAFLD1	Phase III	NCT04197479	1143 (actual)	US	52 weeks	Completed	https://clinicaltrials.gov/study/NCT04197479?term=NCT04197479&rank=1 [95]
	Results of primary endpoint									
	Safety and tolerability: Any TEAE (%): open-label resmetirom 100 mg, 86.5%; resmetirom 100 mg, 86.1%; resmetirom 80 mg, 88.4%; placebo, 81.8%. ≥ 1 drug-related TEAE (%): open-label resmetirom 100 mg, 36.8%; resmetirom 100 mg, 36.7%; resmetirom 80 mg, 34.9%; placebo, 24.2%. ≥ 1 serious TEAE (%): open-label resmetirom 100 mg, 4.1%; resmetirom 100 mg, 7.4%; resmetirom 80 mg, 5.8%; placebo, 6.3%									
Resmetirom	THR-β agonists	Metabolic	MAESTRO-NASH	Phase III	NCT03900429	1759 (actual)	Global	52 weeks	Completed	https://clinicaltrials.gov/study/NCT03900429 [11]
	Results of primary endpoint									
	MASH resolution with no worsening of fibrosis (%): resmetirom 80 mg, 25.9%; resmetirom 100 mg, 29.9%; placebo, 9.7% (*p* < 0.001). Fibrosis improvement ≥ 1 stage with no worsening of MASLD activity score (%): resmetirom 80 mg, 24.2%; resmetirom 100 mg, 25.9%; placebo, 14.2% (*p* < 0.001)									
Semaglutide	GLP-1RA	Metabolic		Phase II	NCT02970942	320 (actual)	Global including Japan	72 weeks	Completed	https://clinicaltrials.gov/study/NCT02970942 [162]
	Results of primary endpoint									
	MASH resolution without worsening of fibrosis (%): semaglutide 0.1 mg, 40.4%; semaglutide 0.2 mg, 35.6%; semaglutide 0.4 mg, 58.9%; placebo, 17.2%									
Semaglutide	GLP-1RA	Metabolic		Phase II	NCT03987451	71 (actual)	Global (other than Japan)	61 weeks	Completed	https://clinicaltrials.gov/study/NCT03987451 [163]
	Results of primary endpoint									
	≥ 1 stage liver fibrosis improvement with no worsening of MASH (%): semaglutide 2.4 mg, 10.6%; placebo, 29.2%									
Semaglutide	GLP-1RA	Metabolic	ESSENCE	Phase III	NCT04822181	1205 (actual)	Global including Japan	72 weeks	Active, not recruiting	https://clinicaltrials.gov/study/NCT04822181 [10]
	Results of primary endpoint									
	Resolution of steatohepatitis with no worsening of fibrosis: semaglutide 2.4 mg, 62.9% vs placebo, 34.3% (EDP 28.7 percentage points; 95% CI 21.1–36.2; *p* < 0.001). ≥ 1-stage improvement in liver fibrosis with no worsening of steatohepatitis: semaglutide 2.4 mg, 36.8% vs placebo, 22.4% (EDP 14.4 percentage points; 95% CI 7.5–21.3; *p* < 0.001)									
Tirzepatide	GLP-1RA	Metabolic	SYNERGY-NASH	Phase II	NCT04166773	190 (actual)	Global, including Japan	52 weeks	Completed	https://clinicaltrials.gov/study/NCT04166773 [114]
	Results of primary endpoint									
	Absence of MASH with no worsening of fibrosis: Tirzepatide 5 mg, (51.84%); 10 mg (63.13%); 15 mg (73.92%); placebo (12.62%)									
Tirzepatide	GLP-1RA	Metabolic	SYNERGY-OUTCOMES	Phase III	NCT07165028	4500 (estimated)	Global, including Japan	224 weeks	Recruiting	https://clinicaltrials.gov/study/NCT07165028
Efinopegdutide	GLP-1RA	Metabolic		Phase IIa	NCT04944992	145 (actual)	Global	24 weeks	Completed	https://clinicaltrials.gov/study/NCT04944992?term=NCT04944992&rank=1 [113]
	Results of primary endpoint									
	Mean relative reduction in liver fat content (LSM, 90% CI) efinopegdutide, 72.7% (66.8–78.7) vs semaglutide, 42.3% (36.5–48.1)									
Survodutide	GLP-1RA	Metabolic		Phase II	NCT04771273	293 (actual)	Global, including Japan	48 weeks	Completed	https://clinicaltrials.gov/study/NCT04771273 [115]
	Results of primary endpoint									
	Histological improvement from baseline: survodutide 2.4 mg, 38.7%; 4.8 mg, 63.8%; 6.0 mg, 55.8%; placebo, 15.2%									
Survodutide	GLP-1RA	Metabolic	LIVERAGE	Phase III	NCT06632444	1800 (estimated)	Global, including Japan	365 weeks	Recruiting	https://clinicaltrials.gov/study/NCT06632444
PXL065	PPAR agonists	Metabolic	DESTINY-1	Phase II	NCT04321343	117 (actual)	US	36 weeks	Completed	https://clinicaltrials.gov/study/NCT04321343?term=PXL065&rank=2#study-overview [126]
	Results of primary endpoint									
	Relative change in liver fat content at Week 36 (LSM [SE]): PXL065 7.5 mg, −22.9% (7.10); 15 mg, −18.6% (6.90); 22.5 mg, −21.3% (6.45); placebo, + 2.4% (6.62)									

*ADA* anti-drug antibodies, *AE* adverse event, *ALP* alkaline phosphatase, *ALT* alanine aminotransferase, *ARB* angiotensin II receptor blocker, *ASBT* apical sodium-bile acid transporter, *ASK1* apoptosis signal-regulating kinase 1, *AST* aspartate aminotransferase, *BID* twice a day, *CCR2* C–C motif chemokine receptor 2, *CCR5* C–C motif chemokine receptor 5, *CVC* cenicriviroc, *CI* confidence interval, *COX* cyclooxygenase, *DGAT2* diacylglycerol O-acyltransferase 2, *EDP* estimated difference, *FC* fold change, *FGFR1c*-*KLB* fibroblast growth factor receptor 1c–klotho beta, *FXR* farnesoid X receptor, *Gal*-*3* Galectin-3, *GGT* gamma-glutamyl transferase, *GLP*-*1RA* glucagon-like peptide-1 receptor agonist, *GR* glucocorticoid receptor, *Hcy* homocysteine, *IL*-*1β* interleukin-1 beta, *KLB* β-klotho, *LDL* low-density lipoprotein, *LSM* least square mean, *MASH *metabolic dysfunction-–associated steatohepatitis, *MASLD* metabolic dysfunction-associated steatotic liver disease, *MR* mineralocorticoid receptor, *MRI*-*PDFF* magnetic resonance imaging-proton density fat fraction, *NAS* NAFLD activity score, *Padj* adjusted *p* value, *PFOR* pyruvate:ferredoxin oxidoreductase, *PK* pharmacokinetics, *PPAR* peroxisome proliferator-activated receptor, *Q4W* every 4 weeks, *QW* once every week, *SAE* serious adverse event, *SD* standard deviation, *SE* standard error, *SEL* selonsertib, *SIM* simtuzumab, *SGLT2* sodium-glucose cotransporter 2, *TEAE* treatment-emergent adverse event, *TXR* tropifexor, *THR*-*β* thyroid hormone receptor-beta, *11β*-*HSD1* 11β-hydroxysteroid dehydrogenase type 1, *UK* United Kingdom, *US* United States

### Preventing or reversing fibrogenesis

Several agents targeting fibrogenesis have been investigated in MASLD/MASH. The cyclophilin inhibitor rencofilstat (ALTITUDE trial, NCT05461105) demonstrated potential anti-fibrotic efficacy with improved liver function [130]. The TANDEM study (NCT03517540) showed that the tropifexor-cenicriviroc combination, targeting dual inflammatory and metabolic pathways, improved fibrosis and achieved steatohepatitis resolution in MASH patients [131]. The CENTAUR trial (NCT02217475) showed cenicriviroc monotherapy improved fibrosis without worsening MASH over a year and provided sustained benefits over 2 years in advanced fibrosis [132, 133]. Emerging strategies under evaluation include elafibranor, selonsertib, aldafermin, PF-06865571, losartan, cotadutide, GB1211, and nitazoxanide, while an erugliflozin trial is currently ongoing (Table 2).

### Reducing hepatic inflammation

Several agents have been investigated for their potential to reduce hepatic inflammation in MASLD/MASH. CM-101, a novel anti-inflammatory agent targeting C-C motif chemokine ligand 24, showed safety in a phase I trial (NCT06037577) [134]. Cyclooxygenase inhibitors such as salsalate and aspirin demonstrated anti-inflammatory effects in MASLD over 6 months (NCT03222206 and NCT04031729) [135]. Miricorilant, a dual glucocorticoid receptor/mineralocorticoid receptor modulator, is being evaluated in the ongoing phase IIb MONARCH trial following safety trials. Other agents, including carbocisteine, MN-001, and diacerein, reflect varied anti-inflammatory strategies (Table 2).

### Improving metabolic regulation

Multiple investigational agents targeting metabolic dysregulation in MASLD/MASH have been explored. Farnesoid *X* receptor agonists (obeticholic acid, EDP-305), THR-β agonists (ALG-055009), and PPAR agonists (saroglitazar) showed potential in improving metabolic parameters (Table 2). Additional strategies include CD3-targeting (OKT3), homocysteine-lowering supplements, cofactor therapy, and inhibition of 11β-hydroxysteroid dehydrogenase type 1 or apical sodium-dependent bile acid transporter, reflecting a broad effort to modulate lipid metabolism (Table 2).

Novel therapies targeting fibrosis, inflammation, and metabolic dysfunction reflect advancements in the MASLD/MASH treatment landscape, enabling a multifaceted, personalized approach to halt or reverse disease progression.

## MASLD/MASH care pathway: challenges and future directions

### Challenges in MASLD/MASH management

The growing burden of MASLD/MASH imposes a substantial economic and healthcare burden. In Japan, direct medical costs associated with non-alcoholic liver cirrhosis reached ¥29.0 billion in 2014. The annual healthcare expenditure for MASLD/MASH patients between 2011 and 2017 ranged from ¥322,206 to ¥340,399, rising to ¥442,723 for MASH, underscoring the economic value of early intervention [136]. However, current clinical pathways remain fragmented, with inconsistencies in healthcare systems and clinical practice, including suboptimal integration and communication between primary, secondary, and tertiary care levels, leading to delayed referrals and late-stage diagnoses [137]. The absence of standard diagnostic criteria and lack of integration between specialties further complicate early identification and timely treatment initiation [137]. Additionally, NILDA such as VCTE, MRE, and blood-based biomarkers (e.g., FIB-4, ELF, COL4-7S, M2BPGi, and CK-18F) are promising; however, their implementation is limited by cost, accessibility, and insurance coverage, particularly in primary care settings [138]. The lack of standardized, consensus-based treatment guidelines also impairs clinical decision-making and restricts international research collaboration.

The complex interplay between MASLD/MASH and metabolic comorbidities necessitates a shift toward integrated, multidisciplinary care models. Such models have demonstrated cost-effectiveness by improving clinical outcomes, reducing long-term complications, and enhancing patient satisfaction. Implementing multidisciplinary care involving hepatologists, cardiologists, diabetologists, nephrologists, nutritionists, and mental health professionals enables comprehensive management of MASLD/MASH and its comorbidities. Evidence from Japan, the United Kingdom, and other countries supports this approach, showing that tailored metabolic hepatology clinics improved liver enzymes, cardiometabolic parameters, and treatment adherence, while reducing hospital visits and delivering economic benefits [80, 139–141]. Cost-effectiveness analyses also support NILDA-based screening strategies. In the United Kingdom, combining FIB-4 with ELF or transient elastography reduced unnecessary specialist referrals and proved more economical than standard care [142]. In the United States, although screening costs for advanced MASLD were higher, the reduction in long-term healthcare expenditure and an incremental cost-effectiveness ratio of $23,265 to $27,884 per quality-adjusted life-year affirmed their value [143].

### Challenges in MASLD/MASH management: patient perspective

MASLD/MASH presents significant diagnostic and management challenges at the patient level due to its heterogeneity. In Japan, the aging population and high prevalence of non-obese MASH, often accompanied by sarcopenia, highlight the need for non-obesity-focused screening and tailored diagnostic strategies. Furthermore, MASLD/MASH is influenced by a complex interplay of genetic, metabolic, and behavioral factors, necessitating personalized approaches for diagnosis and treatment. While lifestyle modification remains the cornerstone of therapy, adherence is suboptimal, owing to psychosocial, cultural, and economic factors [144]. Moreover, access to NILDA and multidisciplinary care is limited by costs and restrictive reimbursement policies [138].

### Ideal MASLD/MASH care pathway

**Fig. 3:**
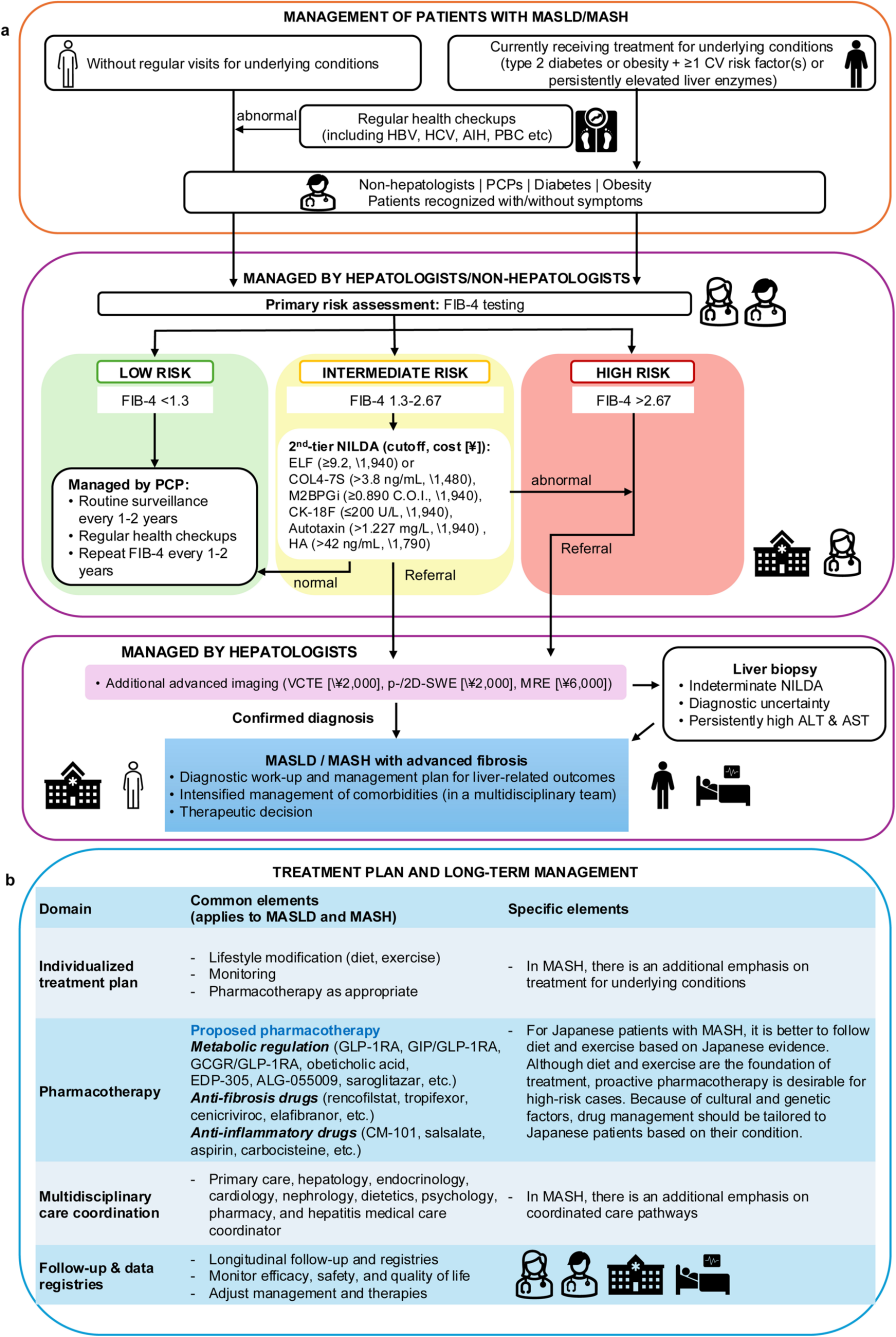
Ideal patient care pathway: (**a**) diagnosis of MASLD/MASH and (**b**) treatment pathway for MASLD/MASH. The figure presents a stepwise diagnostic and management algorithm for routine clinical practice. Alternative causes of liver disease should be excluded before diagnosing MASLD. Initial risk stratification is performed using the FIB-4 index in primary care: low-risk patients (FIB-4 < 1.3) undergo routine follow-up every 1–2 years, intermediate-risk patients (FIB-4 1.3–2.67) proceed to second-line non-invasive testing using blood-based markers and/or imaging, and high-risk patients (FIB-4 > 2.67) are referred to hepatologists for advanced imaging (VCTE, SWE, or MRE) and possible liver biopsy. Management is individualized according to fibrosis stage and disease activity. *AIH* autoimmune hepatitis, *ALT* alanine aminotransferase, *ASBT* apical sodium-bile acid transporter, *AST* aspartate aminotransferase, *CK*-*18F* cytokeratin-18 fragment, *C.O.I* cutoff index, *COL4*-*7S* serum type IV collagen 7S, *CV* cardiovascular, *DPP*-*4i* dipeptidyl peptidase-4 inhibitor, *ELF* enhanced liver fibrosis, *FGF21* fibroblast growth factor 21, *FIB*-*4* fibrosis-4, *GLP*-*1RA* glucagon-like peptide-1 receptor agonist, *HA* hyaluronic acid, *HBV* hepatitis B virus, *HCV* hepatitis C virus, *11β*-*HSD1* 11β-hydroxysteroid dehydrogenase type 1, *LSM* liver stiffness measurement, *MASH* metabolic dysfunction-associated steatohepatitis, *MASLD* metabolic dysfunction-associated steatotic liver disease, *MRE* magnetic resonance elastography, *MRI*-*PDFF* magnetic resonance imaging–proton density fat fraction, *M2BPGi* Mac-2-binding protein glycosylation isomer, *NILDA* non-invasive liver disease assessment, *PBC* primary biliary cholangitis, *PCP* primary care physician, *PPAR* peroxisome proliferator-activated receptor, *SGLT2i* sodium-glucose cotransporter-2 inhibitor, *2D*-*SWE* two-dimensional shear wave elastography, *p*-*SWE* point shear wave elastography, *THR*-*β* thyroid hormone receptor-beta, *VCTE* vibration-controlled transient elastography

An ideal MASLD/MASH care pathway emphasizes early detection, efficient risk stratification, and multidisciplinary management (Fig. 3). Initial assessment should occur in a primary care setting to rule out other causes of steatosis and employ a two-step NILDA strategy. FIB-4 is a robust, non-invasive prescreening tool that reliably excludes advanced fibrosis and effectively prioritizes patients at risk, including older adults, for advanced liver disease. It demonstrates prognostic value for liver-related outcomes and potential utility in monitoring steatohepatitis activity and treatment response. Low-risk individuals (FIB-4 < 1.3 or otherwise healthy) are managed with routine surveillance in non-specialist settings every 1–2 years. Those with indeterminate FIB-4 (1.3–2.67) undergo advanced, second-tier testing with blood-based fibrosis markers (e.g., ELF, COL4-7S, M2BPGi, CK-18F, autotaxin, or hyaluronic acid) to refine risk and avoid unnecessary referrals. High-risk patients (FIB-4 > 2.67 or abnormal second-tier tests) are referred directly to hepatologists for further evaluation with advanced imaging NILDA, such as VCTE, 2D-SWE, point SWE, or MRE to determine treatment strategy and follow-up. At this third tier, advanced imaging NILDA and liver biopsy may be selected according to clinical context, resource availability, and diagnostic needs. This tiered diagnostic strategy improves diagnostic precision, reduces false positives, and lowers costs by minimizing unnecessary specialist referrals. Since imaging devices such as VCTE and MRE are not yet widespread in Japan, selecting cases with suspected progression of liver fibrosis by using blood-based fibrosis markers can contribute to more efficient use of healthcare resources. Upon specialist evaluation, treatment decisions should be individualized based on clinical factors. As new targeted pharmacotherapies become available, treatment decisions may increasingly rely on the patient’s pathophysiological profile, as enabled by advancements in genomics, metabolomics, and NILDA.

Lifestyle and dietary modifications remain the foundation of MASLD management. However, as noted above, robust evidence supporting diet and exercise interventions in the Japanese population remains limited, and adherence to lifestyle modification is often suboptimal due to psychosocial, cultural, and economic factors [144]. Nevertheless, given the differences in genetic background and cultural dietary patterns compared with Western populations, it is essential to establish diet and exercise strategies suited to Japanese patients and to accumulate population-specific evidence. Studies in Japanese patients suggest that exercise and dietary interventions can improve hepatic steatosis and liver-related parameters, although effects on fibrosis are variable. Aerobic and resistance exercise have been shown to improve hepatic steatosis in the majority of MASLD protocols evaluated [145], and exercise-based interventions in Japanese cohorts have demonstrated reductions in liver fat, liver stiffness, and improvements in inflammatory markers and metabolic profiles [104]. However, some studies indicate that lifestyle interventions do not uniformly result in fibrosis improvement [103, 146]. In patients with advanced fibrosis, delaying pharmacological intervention while awaiting lifestyle-induced improvement may not be feasible; therefore, pharmacological therapy should be considered when appropriate.

In Japan, with a basis of lifestyle modification, the therapeutic landscape for MASLD management is evolving. Although resmetirom has been approved in Western countries, it has not yet been submitted for approval in Japan. On the other hand, semaglutide is currently under regulatory review and is expected to be available soon [147]. Furthermore, as international clinical trials including Japanese populations are currently underway, agents such as tirzepatide and survodutide may also become available, pending ongoing clinical and regulatory evaluation in the coming years.

As described above, a characteristic feature of MASLD in Japan is the high proportion of older and non-obese patients. Agents such as GLP-1RA or dual GIP/GLP-1 receptor agonists exert part of their therapeutic effect through weight reduction; hence, care is required when prescribing these agents to older or non-obese individuals to avoid excessive weight loss and prevent sarcopenia. Accordingly, combining pharmacological therapy with appropriately tailored diet and exercise, while closely monitoring body composition, may allow these treatments to be used effectively and safely in typical Japanese patient populations. The ideal patient care model, as shown in Fig. 3, is multidisciplinary, involving coordinated input from physicians, specialists, dietitians, psychologists, pharmacists, and hepatitis medical care coordinators, with the goal of optimizing liver health and addressing systemic metabolic dysfunction. Ongoing real-world data collection and long-term follow-up are critical for ensuring safety, efficacy, and improved quality of life, marking a paradigm shift toward proactive, data-driven MASLD/MASH management.

## Conclusion/summary

MASLD/MASH is a metabolic dysfunction; therefore, its liver-based evaluation is important, rather than relying on anthropometric indicators such as BMI, particularly in non-obese Japanese individuals. In this population, fibrosis risk may be underestimated without appropriate NILDA. Older adults with comorbidities are at increased risk of advanced fibrosis, cirrhosis, or HCC, underscoring the need for hepatologist referral in patients with high NILDA scores/markers. As the global burden of MASLD/MASH rises, risk stratification strategies must be adapted to regional healthcare settings. For low-risk individuals, long-term management should focus on surveillance and lifestyle modification, with pharmacological interventions as needed. For high-risk patients, early specialist involvement and aggressive intervention with newly developed drugs are expected to prevent disease progression. In Japan, a multidisciplinary care model that integrates early diagnosis and coordinated management across specialties may be crucial for reducing the progression of MASLD to MASH, particularly in aging and high-risk populations.

## Abbreviations

ALT: Alanine aminotransferase
AST: Aspartate aminotransferase
AUROC: Area under the receiver operating characteristic
BMI: Body mass index
CAP: Controlled attenuation parameter
CI: Confidence interval
CK-18F: Cytokeratin-18 fragment
CLEIA: Chemiluminescent enzyme immunoassay
COL4-7S: Serum type IV collagen 7S
CVD: Cardiovascular disease
DPP-4i: Dipeptidyl peptidase-4 inhibitor
ELF: Enhanced liver fibrosis
FDA: Food and Drug Administration
FFA: Free fatty acid
FGF21: Fibroblast growth factor 21
FIB-4: Fibrosis-4
FLI: Fatty liver index
*GCKR*: Glucokinase regulator
GLP-1RA: Glucagon-like peptide-1 receptor agonist
HCC: Hepatocellular carcinoma
*HSD17B13*: Hydroxysteroid 17-beta dehydrogenase 13
HSI: Hepatic Steatosis Index
IL-6: Interleukin-6
LSM: Liver stiffness measurement
MASLD: Metabolic dysfunction-associated steatotic liver disease
MASH: Metabolic dysfunction-associated steatohepatitis
*MBOAT7*: Membrane-bound O-acyltransferase domain-containing 7
MRE: Magnetic resonance elastography
MRI: Magnetic resonance imaging
MRI-PDFF: MRI-estimated proton density fat fraction
M2BPGi: Mac-2 binding protein glycosylation isomer
NAFLD: Non-alcoholic fatty liver disease
NAS: NAFLD activity score
NASH: Non-alcoholic steatohepatitis
NFS: NAFLD fibrosis score
NILDA: Non-invasive liver disease assessment
NPV: Negative predictive value
OR: Odds ratio
PNPLA3: Patatin-like phospholipase domain-containing protein 3
PPAR: Pan-peroxisome proliferator-activated receptor
SGLT2i: Sodium-glucose cotransporter 2 inhibitor
SWE: Shear wave elastography
THR-β: Thyroid hormone receptor-β
*TM6SF2*: Transmembrane 6 superfamily member 2
TNF-α: Tumor necrosis factor alpha
T2D: Type 2 diabetes
VCTE: Vibration-controlled transient elastography
